# Selective separation of light rare-earth elements by supramolecular encapsulation and precipitation

**DOI:** 10.1038/s41467-022-32178-3

**Published:** 2022-08-03

**Authors:** Joseph G. O’Connell-Danes, Bryne T. Ngwenya, Carole A. Morrison, Jason B. Love

**Affiliations:** 1 EaStCHEM School of Chemistry, University of Edinburgh, Edinburgh, EH9 3FJ UK; 2 School of Geosciences, University of Edinburgh, Edinburgh, EH9 3FE UK

**Keywords:** Molecular capsules, Sustainability, Coordination chemistry

## Abstract

Supramolecular chemical strategies for Rare Earth (RE) element separations are emerging which amplify the small changes in properties across the series to bias selectivity in extraction or precipitation. These advances are important as the REs are crucial to modern technologies yet their extraction, separation, and recycling using conventional techniques remain challenging. We report here a pre-organised triamidoarene platform which, under acidic, biphasic conditions, uniquely and selectively precipitates light RE nitratometalates as supramolecular capsules. The capsules exhibit both intra- and intermolecular hydrogen bonds that dictate selectivity, promote precipitation, and facilitate the straightforward release of the RE and recycling of the receptor. This work provides a self-assembly route to metal separations that exploits size and shape complementarity and has the potential to integrate into conventional processes due to its compatibility with acidic metal feed streams.

## Abstract (web summary)

The separation of the rare-earth elements is a significant scientific challenge. Here, the authors report the selective precipitation of the light rare-earths as supramolecular capsules from acidic, industrially relevant, mixed-metal solutions.

## Introduction

Supramolecular chemistry concepts such as self-assembly, non-covalent interactions (NCIs), and pre-organisation are increasingly evident in hydrometallurgical extraction and recycling processes^1–4^. These concepts are also relevant to new procedures that separate the rare-earth (RE) elements which are critical elements and crucial for modern, sustainable, and green technologies^5–10^. While diglycolamide ligands are well-known heavy RE extractants, it was only recently appreciated that NCIs between nitrate counter-anions and water clusters within a trefoil-knot organic structure are responsible for selectivity^11^. Furthermore, structurally pre-organised diglycolamide-decorated resorcinarenes extracted selectively heavy REs from nitric acid, reportedly forming bowl-shaped, solvated species in the organic phase^12^. Multivalency principles were exploited in the favourable isolation of heavy RE metal-organic framework (MOF) structures of olsalazine ligands^13^. Similarly, low-coordinate, heavy RE MOFs were formed selectively from thiophene-dicarboxylates and urea solvents depending on solvent and anion structure-directing effects^14^. Significantly, the self-assembly of tetrahedral RE_4_(L)_4_ cages showed a self-sorting, multivalence mechanism in organic solvents that favoured heavy over light RE cages^15^. Selective precipitation of RE complexes was also seen from organic or mildly acidic solutions using tripodal ligands that bias monomer-dimer solution equilibria, offering significant enhancement of separation factors (SFs) between selected pairs of REs, for example dysprosium over neodymium^16,17^.

These advances rely on the coordination of ligands to the RE, thereby dictating selectivity towards the heavy REs due to their smaller ionic radii and enhanced Lewis acidity. In contrast, ionic liquids have been shown to preferentially extract the light REs as dynamic supramolecular nitratometalate/ionic liquid assemblies, albeit from concentrated-nitrate aqueous phases and not from the acidic solutions necessary for RE leaching^18–20^. In order to target the separation of light REs, the formation of RE metalates for anion-transport mechanisms is clearly advantageous. However, RE metalates are not formed in aqueous phases^21^, and their speciation in organic phases is ill-defined and extremely difficult to control. Nevertheless, pre-organised receptors can provide supramolecular host environments for these complex anion guests. A tripodal hexapyridyl triazine was reacted with RE chlorides and nitrates to form host:guest complexes of RE chlorido- and nitratometalates; a 1:1 bowl-shaped environment for chloride was reported whereas a completely encapsulated 1:2 complex formed with nitrate^22,23^. Supramolecular π-anion interactions were evident along with electrostatic interactions due to protonation of the receptor, but poor extraction and negligible selectivity was seen from aqueous nitric acid. Similarly, a pre-organised arene/quinoline-ether tripodal receptor formed a 1:1 host:guest complex with RE(NO_3_)_3_(MeOH)_3_ in methanol through ether-alcohol hydrogen bonding interactions^24^. Pre-organised hexa-substituted arene platforms are well-known to self-assemble into supramolecular tennis-ball structures^25,26^, and can encapsulate simple anions such as fluoride, nitrate, and arsenate^27–29^.

We anticipated that hexa-substituted arenes with amide appendages would have the potential to form capsular compounds with RE metalates and that this would be favoured due to the amide N-H hydrogen bonding interior along with amide O-atom sites of protonation^30^. Here we show that an amide-functionalised hexa-substituted arene acts as a highly selective precipitant for light RE elements from 4–8 M nitric acid through the formation of RE nitratometalate supramolecular capsules.

## Results

### Lanthanum capsule precipitation and characterisation

**Fig. 1:**
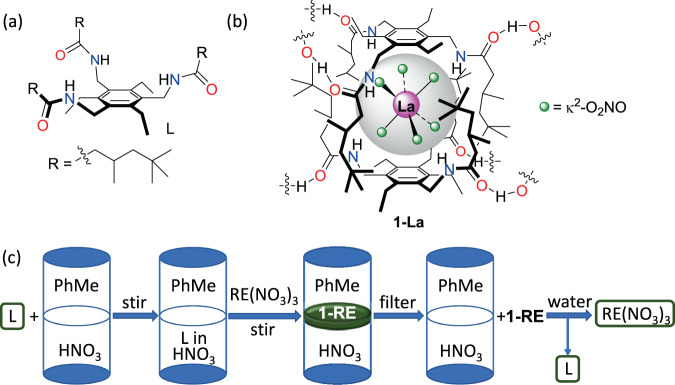
Encapsulation and precipitation of the rare-earth elements (REs). **a** The tripodal amido-arene L used in this study, **b** the capsular hexanitratometalate complex, [{La(κ^2^-NO_3_)_6_}⊂(H_3_L_2_)]_n_ **1-La**, **c** a schematic of the precipitation of rare-earths by L from a biphasic nitric acid/toluene mixtures and its stripping using a polar protic solvent such as water.

The tripodal amido-arene L (Fig. 1) was prepared and while only partially soluble in toluene and insoluble in nitric acid, dissolves into the aqueous phase of a biphasic mixture of toluene and 8 M HNO_3_. Addition of La(NO_3_)_3_ to this biphasic mixture with vigorous stirring over 24 h causes **1-La** to precipitate at the interface (Fig. 1). Analysis of both aqueous and organic phases by ICP-MS shows that complete precipitation of **1-La** occurs.

**Fig. 2:**
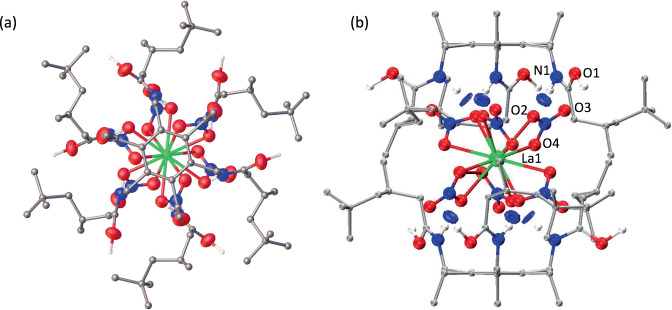
X-ray crystal structure and DFT NCI analysis of 1-La [{La(NO_3_)_6_}⊂(H_3_L_2_)]_n_. **a** X-ray crystal structure (top-down view). For clarity, all hydrogen atoms except those involved in hydrogen bonding and a disorder component of the amide arm are omitted (where shown, thermal displacement ellipsoids are drawn at 50% probability). N-H and O-H hydrogen atoms were located in the difference Fourier map and (O1)H is 50% occupied on a crystallographic special position. Atom colours: La = green; oxygen = red; nitrogen = blue; carbon = silver; hydrogen = grey. **b** DFT structure showing non-covalent interactions. NCI attractive hydrogen bond interactions are represented as blue disks. Atom colours: La = green; oxygen = red; nitrogen = blue; carbon = silver; hydrogen = grey.

The La-containing precipitate **1-La** was isolated by filtration and slurried in acetonitrile, from which crystals formed over 4 months. The X-ray crystal structure displays the highly symmetrical supramolecular capsule [{La(NO_3_)_6_}⊂(H_3_L_2_)]_n_ in which the hexanitratometalate La(NO_3_)_6_^3−^ is encapsulated by two protonated amido-arene receptors (Fig. 2). These receptors adopt *ababab* configurations similar to those seen for “flexi-ball” and related supramolecular structures derived from statically geared, hexa-alkylarene platforms^26,31^. The La(NO_3_)_6_^3−^ anion is twelve coordinate with the nitrates asymmetrically κ^2^-bound (La1-O4 = 2.6593(17) Å, La1-O2 = 2.6370(17) Å). The capsular structure is reinforced by intramolecular hydrogen bonding between the amide N-H and a single nitrato O-atom that is coordinated to La (N1(H)---O2 = 2.814(3) Å) and are shown by NCI calculations to be the dominant intra-capsule attraction (see Supplementary Information Fig. 1). These interactions result in the orientation of the pseudo-octahedral nitratometalate such that the apical nitrate oxygen atom O3 occupies space between the interdigitating arms of the tripodal receptors. As such, the structure of the interior of the capsule complements that of the hexanitratometalate like those seen in the encapsulation of metalates by self-assembled coordination cages and functional molecular flasks^32–36^. Each amide oxygen of each receptor L is ½ protonated, resulting in a total of three protons per capsule which balances the charge of the trianionic metalate. This results in a polymeric, solid-state structure through hydrogen bonding between protonated amide oxygen atoms of adjacent molecules (O1(H)---O1’ = 2.439(3) Å) (see the Supplementary Information Fig. 2 for the extended structure). These short inter-capsule hydrogen bonds are shown by quantum theory atoms in molecules (QTAIM)^37^ analysis of the promolecular electron density of the DFT optimised structure to be about 3.5 times stronger than the intra-capsule N-H---O bonds (see Supplementary Information Fig. 3). Thus, it is evident that the protonated receptor presents a geometry that compliments the hexanitratometalate through efficient intra-capsule hydrogen bonding and is also designed to present strong inter-capsule interactions that ultimately leads to precipitation.

Powder X-ray diffraction analysis of the bulk precipitate of **1-La** shows excellent correlation with the calculated pattern derived from the single-crystal structure and confirms that the bulk precipitate and single-crystals are structurally coherent (Supplementary Information Figs. 4 and 5). The Raman spectrum of **1-La** shows absorptions at 1055 and 1039 cm^−1^ assigned to La-O_2_(NO) asymmetric and symmetric stretches (Supplementary Information Fig. 6). Evidence of protonation at the carbonyl oxygen atom is provided by a shift in the C=O stretch from 1638 cm^−1^ in L to 1576 cm^−1^ in **1-La** and the IR spectrum shows absorptions at 3223 and 3069 cm^−1^ for hydrogen-bonded N-H and O-H groups that are shifted from those in L (ν(N-H) 3297 cm^−1^) (Supplementary Information Fig. 7). Mass spectra (ESI/MALDI/LDI) show only ions related to “half-capsule” compounds such as La(NO_3_)_2_(L)^+^ (*m*/*z* = 932.46). The limited solubility of **1-La** in acetonitrile also inhibits the recording of any useful NMR spectra. It is evident from the accumulated data that the bulk material that precipitates from the biphasic mixture is capsular in nature.

### Selective rare-earth precipitation

**Fig. 3:**
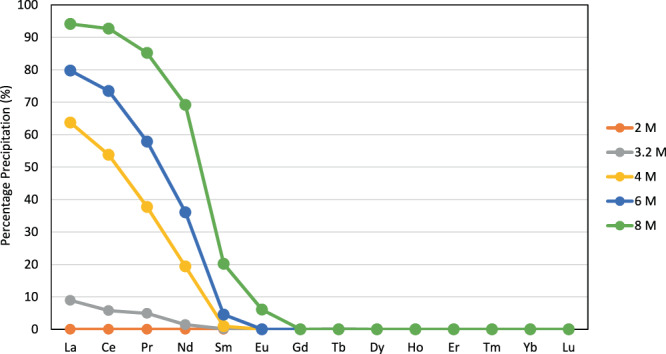
Precipitation of REs by the tripodal amido-arene L. Precipitation arising from a 0.0025 M mixed-RE solution in 2 to 8 M HNO_3_/toluene equal-volume biphasic mixture after the addition of 3.5 mmol L (5-fold excess L relative to metal) at 298 K.

Significantly, RE precipitations by L from mixed-metal RE(NO)_3_ solutions are selective. Precipitation of La to Lu (excluding Pm) with excess L from 8 M HNO_3_/toluene follows a sigmoidal trend, with excellent uptake of La to Nd (95–70%) compared with negligible/zero uptake of Eu to Lu (<10–0%) (Fig. 3). To our knowledge, this is the first example of selective metal separation by supramolecular encapsulation of any metalate, not just the REs. The structures of the precipitates **1-Pr** and **1-Nd** were evaluated by PXRD and show a very similar pattern to that of **1-La**, albeit with shifted 2*θ* values as expected for smaller unit cell parameters for capsular complexes of these metals (Supplementary Fig. 8).

**Fig. 4:**
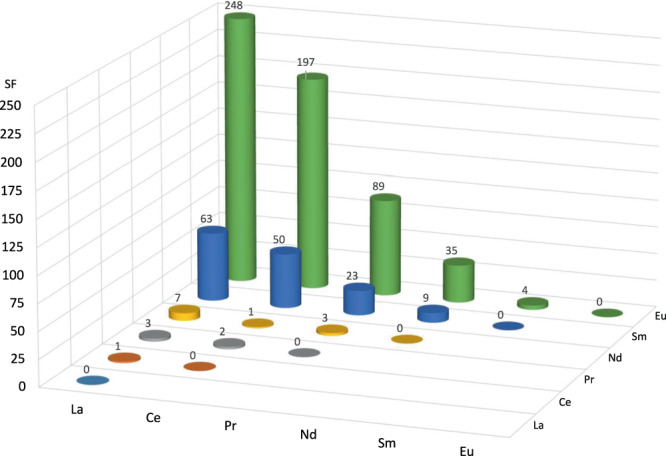
Separation factors (SFs) between REs (La-Eu) on precipitation by L. Determined from precipitations arising from a 0.0025 M mixed-metal solution in 8 M HNO_3_/toluene equal-volume biphasic mixture after the addition of 3.5 mmol L (5-fold excess L relative to metal) at 298 K. Separation factors are calculated from pseudo-distribution ratios, where the metal lost from the aqueous phase (relative to the feed solution) is assumed to be entirely contained in the precipitate.

It is instructive to use (pseudo) SF values to contextualise the selectivity in precipitation even though they are normally derived from distribution coefficients for the partitioning of metals between two solvent phases (Fig. 4). The SF_La/RE_ (where RE = Ce-Eu) at 298 K increase dramatically in magnitude across the series; no SFs were calculated for elements beyond Eu as precipitation is zero within experimental error. While SFs for La/Ce, La/Pr, and La/Nd are similar to the state-of-the-art phosphorus acids in solvent extraction experiments^38^, those involving La/Sm and onwards are larger, from 63 for La/Sm to 248 for La/Eu. As no Dy precipitation is seen the SF_Nd/Dy_ is effectively infinite and therefore greatly exceeds those seen in separations using state-ot-the-art tripodal hydroxypyridone (SF_Dy/Nd_ 213)^17^ and borate (SF_Nd/Dy_ 986) precipitants^39^.

### Effect of variables on precipitation and separation

The concentration of nitric acid is an important factor for both the extent and selectivity of precipitation (Fig. 3). No or little precipitation is seen for concentrations below 4 M, while increasing from 6 to 8 M HNO_3_ increases precipitation of the light REs with a slight shift to the right of the series. Increasing the concentrations of nitric acid to 10 and 12 M causes a further shift to the right of the RE series but these data are compromised due to significant nitration of the toluene solvent (Supplementary Information Fig. 9). This enhanced precipitation behaviour seen at high acid concentrations is unusual for anion transport and precipitation mechanisms for which acid/anion transport is usually competitive and inhibits metal uptake. At 4 M HNO_3_, while precipitation is lower in extent (64% La vs. 94% at 8 M HNO_3_) a marked shift in SFs is evident, becoming infinite beyond Sm (SF_La/Sm_ 180, SF_La/Nd_ 7).

The effect of M:M’ ratio on the amount and selectivity of precipitation was probed for three binary mixtures: La/Nd; La/Dy; Nd/Dy at 5:1; 1:1; 1:5 ratios (Supplementary Information Fig. 10). While the selectivity is essentially unchanged by the presence of an excess of metal, the overall precipitation is enhanced at higher absolute metal concentrations. Furthermore, at metal concentrations a factor of ten lower, negligible precipitation is seen whereas at a factor of ten higher the large quantity of precipitate produced results in significant entrainment of the aqueous phase and erroneous concentration measurements.

Varying the M:L ratio in a La single-metal biphasic mixture results in an increase in precipitation up to *ca*. two equivalents of L, after which little increase in precipitation quantity is seen (Supplementary Information Fig. 11); this supports the observed formulation of the capsular complex.

The presence and identity of the organic phase is crucial. The tripodal amide L is insoluble in HNO_3_ at the concentrations investigated and only dissolves in biphasic mixtures with toluene or heptane; L dissolves in chloroform but, in this case, remains unprotonated in the organic phase. Dissolution of L into 8 M HNO_3_ under biphasic conditions, followed by removal of the toluene phase and addition of La(NO_3_)_3_, results in no precipitation of **1-La**. While the organic phase is requisite for precipitation, the organic/aqueous (o/a) ratio can be very low, with the extent of precipitation of **1-La** similar for o/a ratios from 0.05 (74%) to 1.0 (81%) along with little variation in RE selectivity between **1-RE** (**RE** = La, Nd, Dy; Supplementary Information Fig. 12). This necessity for a non-polar organic phase hints that capsule formation occurs at an oil-water interface similar to that seen for supramolecular microcapsules^40^. Also, the prevalence of *penta-*nitratometalate structures of the heavy REs suggests that structural complementarity between the pre-organised host framework and guest nitratometalates in the supramolecular assembly process is important to selectivity. The formation of higher-order nitratometalates is more likely in a non-polar phase^18^, so reinforcing the importance of the biphasic assembly process.

Finally, the stripping of the metal from **1-La** and the recycling of L is straightforward. The **1-La** precipitate is isolated by filtration and washed with HNO_3_, removing any unprecipitated REs. Subsequent dissolution of the solids in a protic solvent such as methanol causes the capsules to rupture and, on addition of water, L precipitates from solution, leaving a supernatant of La(NO_3_)_3_ in methanolic HNO_3_. Ligand L can be recycled for further use (Supplementary Information Fig. 13). This ease of stripping the RE and recycling of L reflects both the supramolecular nature of the bonding in **1-RE** and the weak basicity of the amide groups, both of which are intrinsic to the receptor design.

The selectivity for the light REs shown by this receptor, its ability to function under highly acidic conditions, and the ease of stripping the RE with recycling of the receptor may contribute to the development of a useful RE separations process. However, these aspects must be balanced by the relatively complex synthesis of the receptor, the potential difficulty in designing a continuous separation process involving precipitation, and the variability of source materials such as RE ores and magnets.

## Methods

All solvents and reagents were used as received from Sigma-Aldrich, Fisher Scientific UK, Alfa Aesar, Acros Organics or VWR International. Deionised water was obtained from a MilliQ purification system.

### Synthesis of tripodal amidoarene (L)

Neat 3,5,5-trimethylhexanoyl chloride (5.64 ml, 29.7 mmol, 3.3 equiv.) was added dropwise to a solution of 1,3,5-tris(aminomethyl)-2,4,6-triethylbenzene (2.41 g, 9.6 mmol) in a mixture of THF (200 ml) and NEt_3_ (4.8 ml). The mixture was stirred at room temperature for 24 h after which the precipitate was filtered and the filtrate evaporated under reduced pressure to give an off-white solid. Recrystallisation from toluene gave 4.0 g, 62% of L as a colourless solid: ^1^H NMR (601 MHz, chloroform-*d*) *δ* 5.21 (t, *J* = 4.5 Hz, 3H), 4.49 (d, *J* = 4.3 Hz, 6H), 2.72 (q, *J* = 7.5 Hz, 6H), 2.19 (dd, *J* = 13.6, 6.2 Hz, 3H), 2.15–2.10 (m, 3H), 1.94 (dd, *J* = 13.6, 7.9 Hz, 3H), 1.27–1.21 (m, 12H), 1.11 (dd, *J* = 13.9, 6.7 Hz, 3H), 1.00 (d, *J* = 6.6 Hz, 9H), 0.93 (s, 27H); ^13^C NMR (126 MHz, CD_3_CN) *δ* 171.64, 143.59, 132.39, 50.28, 45.58, 37.34, 30.56, 29.33, 27.19, 22.61, 22.02, 15.58; FT-IR *ν* = 3304, 2955, 2904, 2869, 1632, 1525, 1494, 1466, 1364; ESI-MS (*m*/*z*) C_42_H_75_N_3_O_3_ [M + Na]+, calcd. 692.570, found 692.571. NMR data are shown in Supplementary Information Figs. 14 and 15.

### Precipitation procedure for mixed-RE solutions

Nitric acid solutions (1–12 M) were prepared by dilution of concentrated nitric acid with ultra-pure deionised water. Mixed-RE solutions (0.0025 M) were prepared by dilution of a 0.1 M stock solution containing La-Lu (no Pm, Sc, Y) metal salts into the prepared nitric acid solutions to give a total aqueous phase volume of 2 ml. Toluene (2 ml) was added to each sample. Solid L (0.35 mmol) was added to a vial along with a magnetic stir bar (the order of addition of RE nitrate and L is not important). The mixture was stirred for 24 h at 298 K at 700 rpm after which the stir bar was removed. Samples were prepared for ICP-MS to measure the metal content remaining in the aqueous phase (compared with the feed solution) to determine metal uptake by L. Samples were diluted by 500x in 2% nitric acid prior to ICP-MS analysis. These procedures were repeated in duplicate.

### Precipitation procedure for binary-mixed-RE solutions varying metal:metal molar ratios

Nitric acid solutions (8 M) were prepared by dilution of concentrated nitric acid with ultra-pure deionised water. Binary-mixed-RE solutions (La/Dy, La/Nd, Nd/Dy) at metal:metal molar ratios of 5:1, 1:5, and 1:1 (0.0125 M and 0.0025 M respectively) were prepared by dilution of 0.1 M stock solutions of La, Nd, and Dy metal salts into the prepared nitric acid solutions to give a total aqueous phase volume of 2 ml. Toluene (2 ml) was added to each sample. Solid L (0.15 or 0.05 mmol, 5-fold excess relative to metal) was added to a vial along with a magnetic stir bar. The mixture was stirred for 24 h at 298 K at 700 rpm after which the stir bar was removed. Samples were prepared for ICP-MS to measure the metal content remaining in the aqueous phase (compared with the feed solution) to determine metal uptake by L. Samples were diluted by 5000x in 2% nitric acid prior to ICP-MS analysis. These procedures were repeated in duplicate.

### Precipitation procedure from single-metal lanthanum solution varying ligand:metal molar ratios

Nitric acid solutions (8 M) were prepared by dilution of concentrated nitric acid with ultra-pure deionised water. La(NO_3_)_3_ (0.0025 M) solutions were prepared by dilution of a 0.1 M stock solution containing lanthanum nitrate into the prepared nitric acid solutions to give a total aqueous phase volume of 2 ml. Toluene (2 ml) was added to each sample. The precipitations were carried out using varying concentrations of L (0.005 mol) which was added to a vial as a solid along with a magnetic stir bar. The mixture was stirred for 24 h at 298 K at 700 rpm after which the stir bar was removed. Samples were prepared for ICP-MS to measure the metal content remaining in the aqueous phase (compared with the feed solution) to determine metal uptake by L. Samples were diluted by 500x in 2% nitric acid prior to ICP-MS analysis.

### Recycling experiments

Nitric acid solutions (8 M) were prepared by dilution of concentrated nitric acid with ultra-pure deionised water. La(NO_3_)_3_ (0.025 M) solutions were prepared by dilution of a 0.1 M stock solution containing lanthanum nitrate into the prepared nitric acid solutions to give a total aqueous phase volume of 4 ml. Toluene (2 ml) was added to each sample. Solid L (0.05 mol) was added to a vial along with a magnetic stir bar. The mixture was stirred for 24 h at 298 K at 700 rpm after which the stir bar was removed. Samples were prepared for ICP-MS to measure the metal content remaining in the aqueous phase (compared with the feed solution) to determine metal uptake by L. Samples were diluted by 1000x in 2% nitric acid prior to ICP-MS analysis. The metal-containing precipitate was collected through filtration and dissolved in methanol (3 ml) and the free ligand precipitated with water (1 ml). The precipitated ligand was collected by filtration and added to a new biphasic solution. The precipitation and analysis procedure were carried out as before.

### X-ray crystal structure of 1-La

Colourless blocks were grown by slow evaporation of a supersaturated solution (10 mM) of **1-La** in acetonitrile over a period of several months. X-ray data were collected at 120 K on an Oxford Diffraction Supernova, Dual, Cu at Zero Atlas diffractometer using Cu-K_α_ radiation (*λ* = 1.5418 Å). The structure was solved by direct methods using ShelXT and refined using a full-matrix least-squares refinement using ShelXL^41,42^, both within the Olex2 (v1.5) software^43^. X-ray data are presented in Supplementary Information Table 1.

### Powder X-ray diffraction

Data for La/Nd/Dy powders were collected using a Bruker D2 phaser diffractometer in reflection geometry with Cu Kα radiation (*λ* = 1.541 Å). A LynxEye position sensitive detector was used to collect data over the 2*θ* range 5–45° for 45 min. Sample preparation involved grinding powder samples, a loading into the recess (1 mm deep) of a zero-background silicon (911) substrate. The La data were analysed using a Pawley fitting routine in the Topas Academic (version 6) software suite.

### ICP-MS analysis

ICP-MS analysis was carried on an Agilent 7800 Single Quadrupole Inductively Coupled Plasma Mass Spectrometer. Samples in 2% nitric acid were taken up by a peristatic pump at a rate of 0.3 rps into a MicroMist nebuliser and a quartz Scott type spray chamber. Argon plasma conditions were 1550 W RF power and gas flows of 15, 1.07, and 0.9 l min^−1^ for plasma, auxiliary, and nebuliser flow, respectively.

### IR and Raman spectroscopy

Raman spectra were recorded on an InVia Renishaw spectrometer at 532 and 785 nm. Fourier transform-infrared (ATR FT-IR) measurements were collected on a Perkin Elmer 65 FT-IR spectrometer over the range 4000–500 cm^−1^.

### Mass spectrometry

ESI-FT-ICR MS measurements of **1-La** in CH_3_CN were recorded in positive-ion mode using the standard Bruker ESI sprayer with a SolariX FTICR mass spectrometer. All mass spectra were analysed using DataAnalysis software. Ions were assigned manually.

### NCI plots and QTAIM analysis

Atomic positions in the crystal structure were optimised using CASTEP17.21^44^, with on-the-fly pseudopotentials and a plane-wavecut off of 750 eV, coupled to the PBE DFT functional and TS dispersion correction scheme^45–47^. Brillouin zone sampling was below 0.05 Å^−1^. Geometry optimisation criteria: energy tolerance = 2 × 10^−5^ eV atom^−1^, max force = 0.05 eV Å^−1^, max atomic displacement = 2 × 10^−3^ Å. Following geometry optimisation, a charge density cube was generated using the CASTEP2CUBE facility and subsequently used to generate the NCI plot using the CRITIC2 code, which was also employed for the QTAIM analysis based on the promolecular electron density derived for the optimised DFT structure^48–50^. The NCI graphical output was processed using VMD1.9.3^51^ to visualise the hydrogen bond interaction 3D isosurface data (presented at reduced electron density *s* = 0.5 au and sign(*λ*2)*ρ* = −0.05–−0.03 au), and Origin19 to present all NCIs as a 2D representation.

## Supplementary information

Supplementary Information

## Data Availability

X-ray data are available free of charge from the Cambridge Crystallographic Data Centre (https://www.ccdc.cam.ac.uk/data_request/cif) under reference number CCDC 2142978. The quantitative metal analyses, IR, Raman, and NMR data are available in the Edinburgh DataShare Repository 10.7488/ds/3419. The authors declare that all other data supporting the findings of this study are available within the paper and its Supplementary Information files.
