# Supplementary material for: Selective separation of light rare-earth elements by supramolecular encapsulation and precipitation
Source: Nat Commun. 2022 Aug 3;13:4497. doi: 10.1038/s41467-022-32178-3 (PMC9349306; doi:10.1038/s41467-022-32178-3)
Supplement: Supplementary file 1 — Supplementary Information [file 41467_2022_32178_MOESM1_ESM.pdf]

Supplementary Information
Selective separation of light rare-earth elements by supramolecular encapsulation and precipitation

Joseph O'Connell-Danes,[1] Bryne T. Ngwenya,[2] Carole A. Morrison,[1] and Jason B. Love1*

[1] EaStCHEM School of Chemistry, University of Edinburgh, Edinburgh, EH9 3FJ, U.K.
[2] School of Geosciences, University of Edinburgh, Edinburgh, EH9 3FE, U.K.

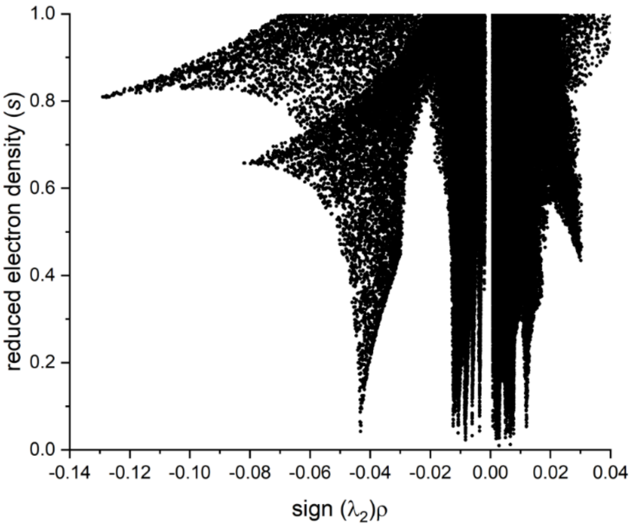

Supplementary Information Figure 1. Two-dimensional NCI plot for **1-La**, derived from the DFT optimised structure electron density, that highlights that the intra-capsule interactions are dominated by the ligand/NO_3^- N-H---O hydrogen bond interactions (indicated by the reduced electron density spike at ca. −0.043 sign (l_2)r. The attractive dispersion interactions (at ca. −0.01 to 0 sign (l_2)r) are largely cancelled out by the repulsive dispersion interactions (at ca. 0 to +0.01 sign (l_2)r). Note the absence of an NCI interaction for the inter-capsule O-H---O interaction highlights the covalent nature of this short and strong hydrogen bond.

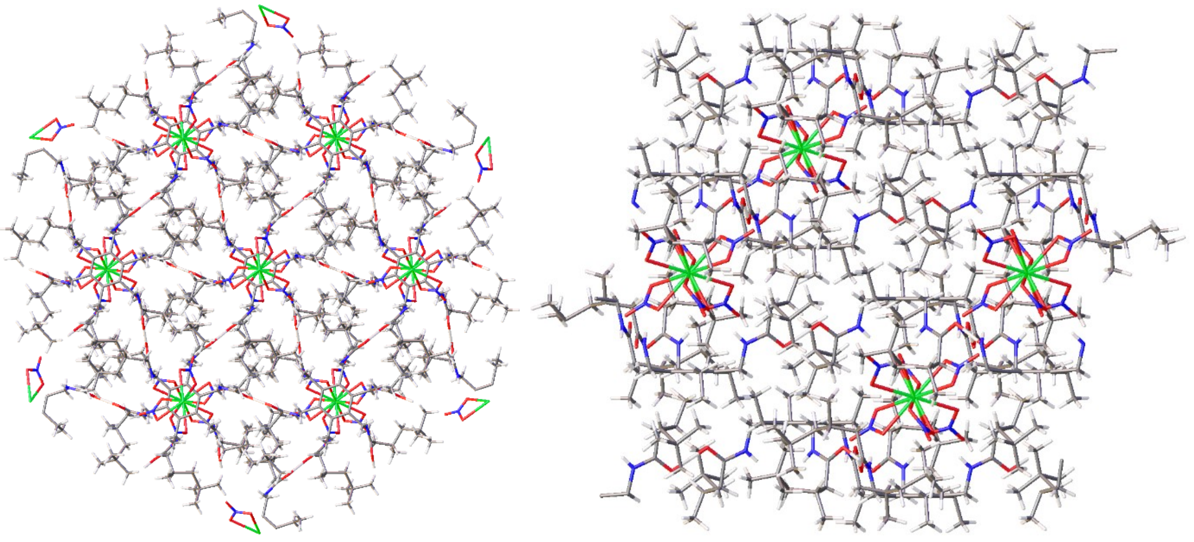

Supplementary Information Figure 2. Top-down and side-on views of the extended crystal structure of **1-La** [{$La(NO_3)_6$}(H_3L_2)]$_n$. N-H and O-H hydrogen atoms were located in the difference Fourier map and (O1)H is 50% occupied on a crystallographic special position.

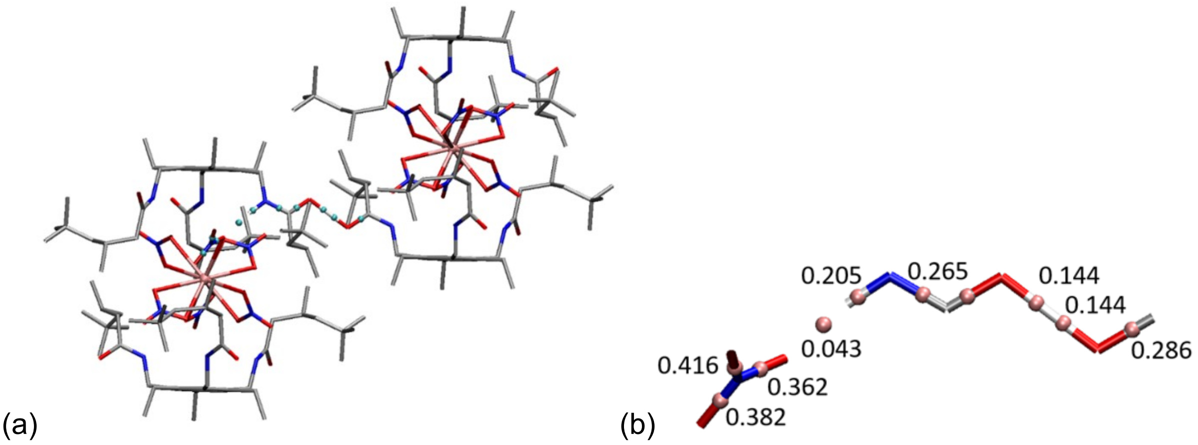

Supplementary Information Figure 3. QTAIM analysis of **1-La**. Calculated BCPs and associated electron densities (eÅ^{-3}) obtained from the promolecular electron density plot of the optimised DFT crystal structure, shown (a) in situ (cyan) and (b) close up (pink). Given that the calculated electron densities can be taken as indicative of bond strength, it is evident that the symmetric O---H---O interaction, with an electron density of 0.144 eÅ^{-3} at each BCP, is just over three times that of the weaker N-H---O bond (at 0.043 eÅ^{-3}). This suggest that that the inter-capsule hydrogen bond linkages are likely to dominate over the intra-capsule bonds, even taking the bond multiplicities into account (per capsule: three strong O-H---O inter-capsule and six weaker N-H---O intra-capsule interactions).

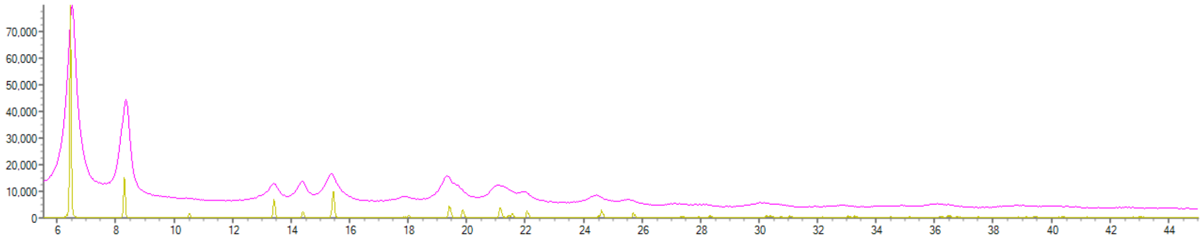

Supplementary Information Figure 4. Comparison of XRPD data collected on a sample of **1-La** (pink) with the theoretical pattern (gold) derived from the single-crystal X-ray data (cif).

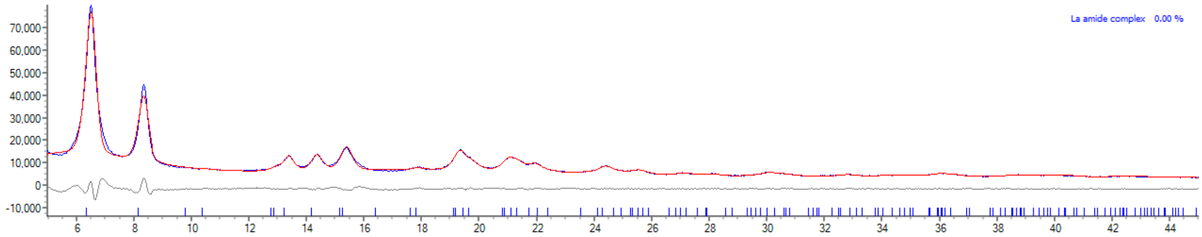

Supplementary Information Figure 5. Pawley refinement of XRPD data collected on **1-La**; observed data (blue), calculated profiled (red), difference profile grey, blue tick marks associated with the complex. The refined XRPD unit cell parameters of **1-La** in space group R-3 were *a* = 21.62(2) Å, *c* = 20.64(3) Å, which are similar to those obtained from the single crystal study (*a* = 21.2897(3) Å, *c* = 21.4144(3) Å,) and confirm the presence of **1-La** in this sample. The differences between the unit cell parameters are due to the measurement of multiple crystallites using XRPD techniques, giving an average of the unit cell parameters, compared to the measurement of a single crystallite using single crystal XRD techniques.

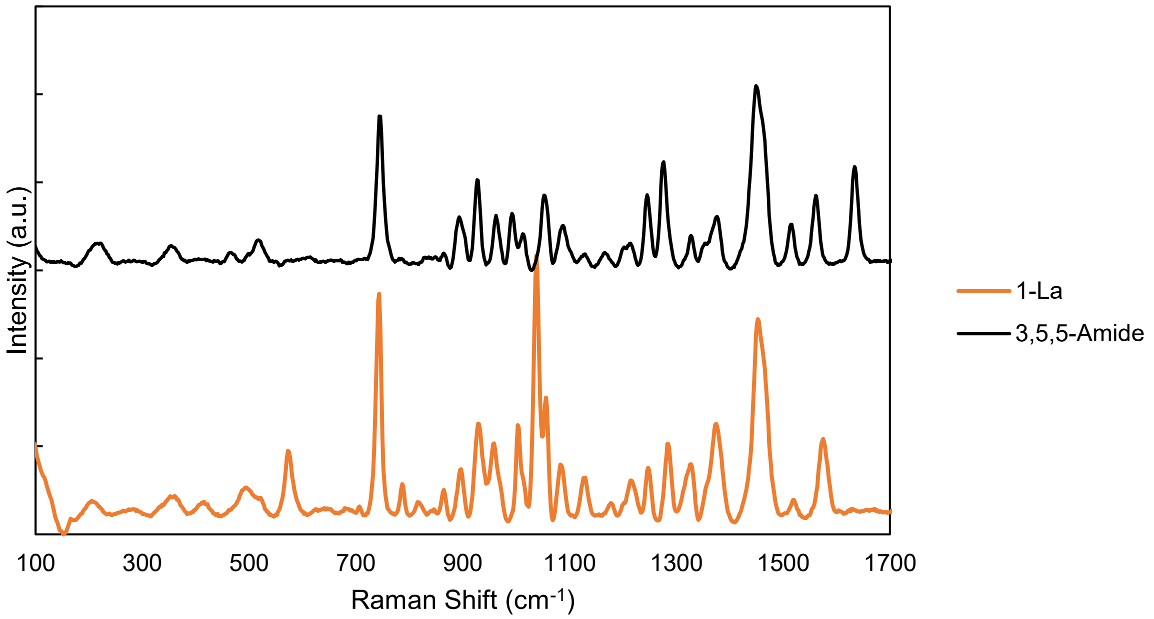

Supplementary Information Figure 6. Comparison of the Raman spectra of the free ligand L (black) with the lanthanum containing precipitate **1-La** (orange).

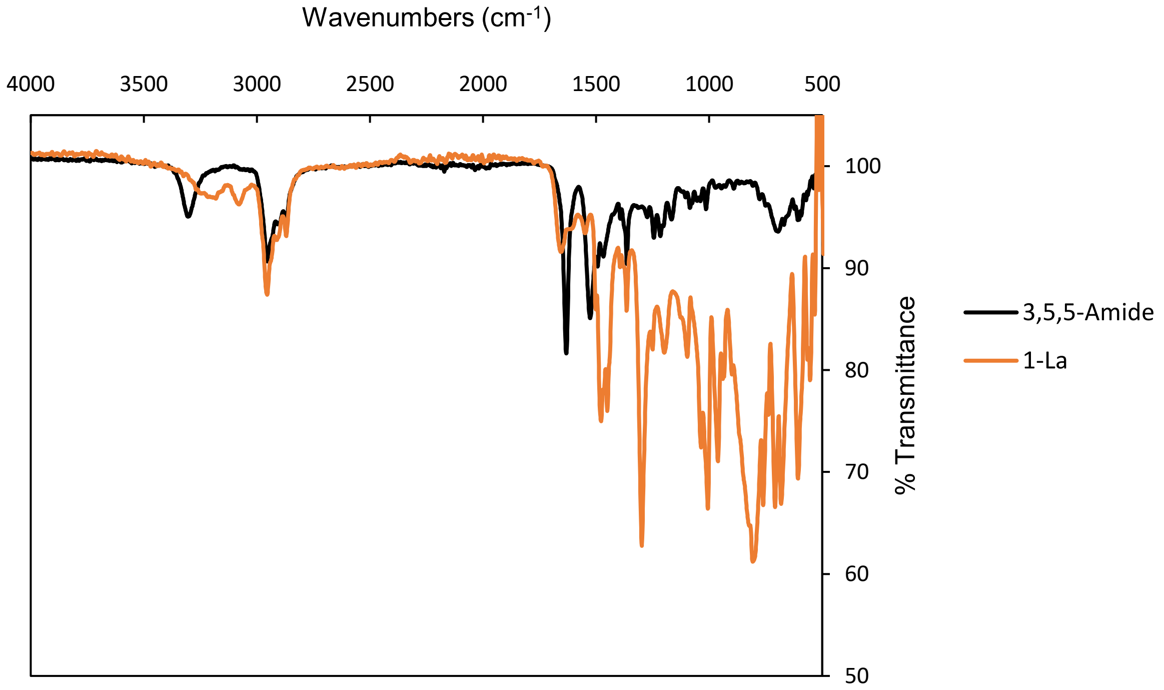

Supplementary Information Figure 7. Comparison of the IR spectra of the free ligand L (black) with the lanthanum containing precipitate **1-La** (orange).

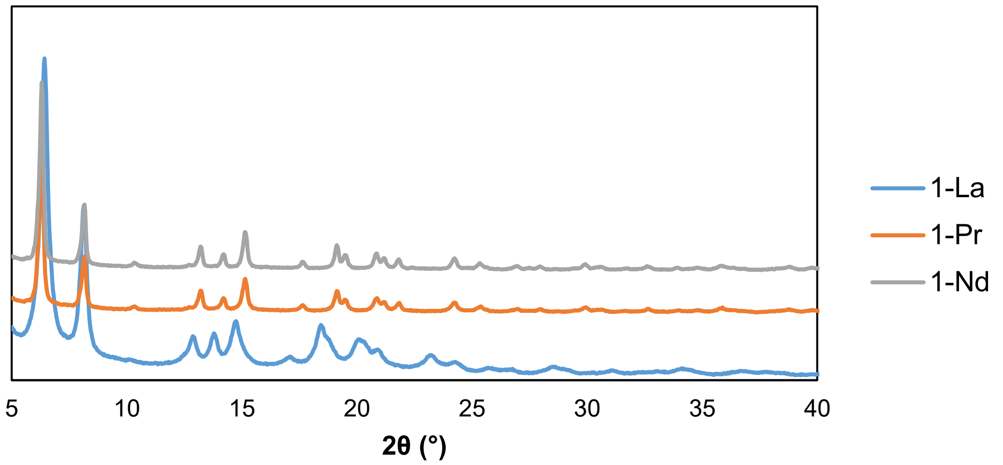

Supplementary Information Figure 8. Comparison of XRPD data collected from isolated samples of **1-La** (blue), **1-Pr** (orange), and **1-Nd** (grey).

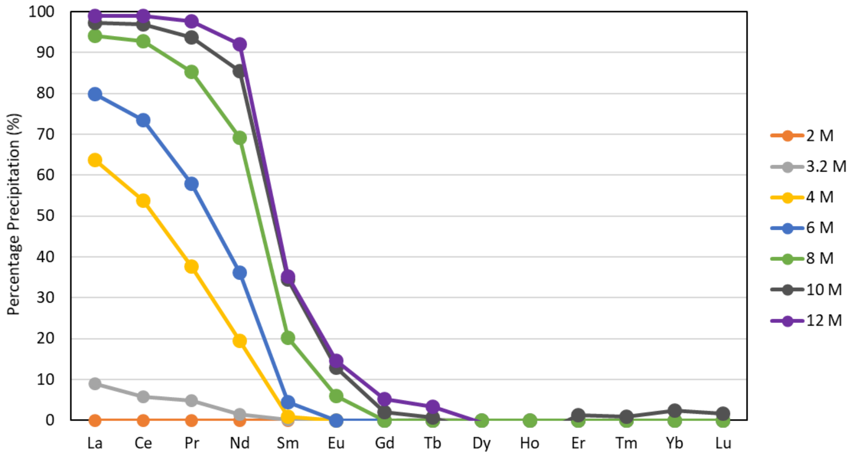

Supplementary Information Figure 9. Precipitation of REs by the tripodal amido-arene L from a 0.0025 M mixed-RE solution in 1 to 12 M HNO_3/toluene equal-volume biphasic mixture after the addition of 3.5 mmol L (5-fold excess L relative to the total metal concentration) at 298 K.

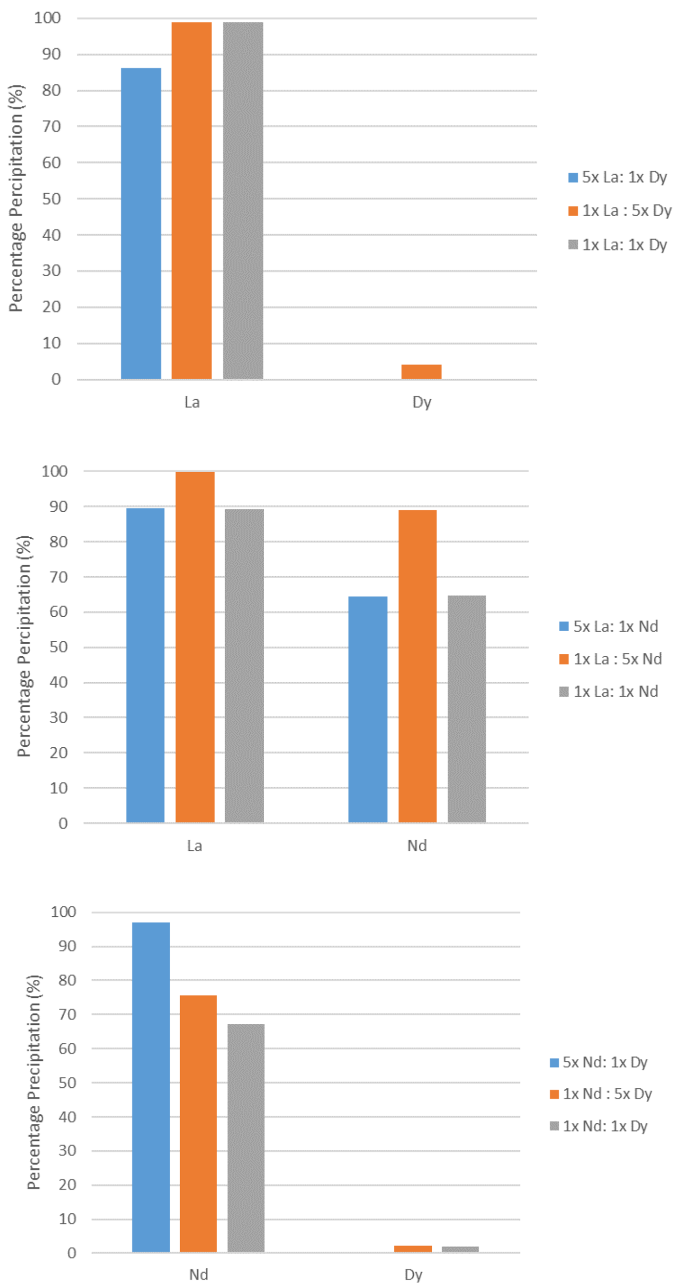

Supplementary Information Figure 10. Precipitation of REs by the tripodal amido-arene L from binary mixed-metal solutions (La/Dy [top], La/Nd [middle], Nd/Dy [bottom]) at metal:metal molar ratios of 5:1, 1:5, and 1:1 (0.0125 M and 0.0025 M respectively) in 8 M HNO_3/toluene equal-volume biphasic mixture after the addition of 0.15 mmol or 0.05 mmol L (5-fold excess L relative to the metal concentration) at 298 K.

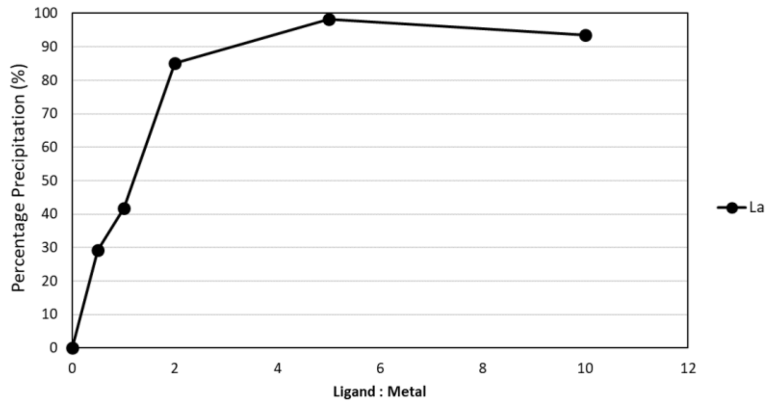

Supplementary Information Figure 11. Precipitation of La by the tripodal amide-arene L from a 0.0025 M $La(NO_3)_3$ solution in 8 M HNO_3 HNO_3/toluene equal-volume biphasic mixture varying the molar ratio of L:M from 0.5 L:1.0 M to 10 L:1.0 M (0.005 mmol to 0.05 mmol) at 298 K.

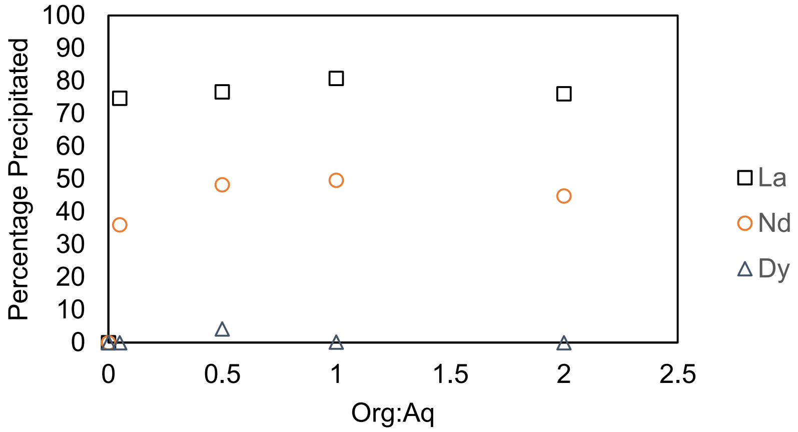

Supplementary Information Figure 12. Precipitation of REs by the tripodal amide-arene L from a 0.0025 M mixed-RE solution in 8 M HNO_3 with a varying volume toluene phase (0.1 to 4 mL) mixture after the addition of 0.075 mmol L at 298 K.

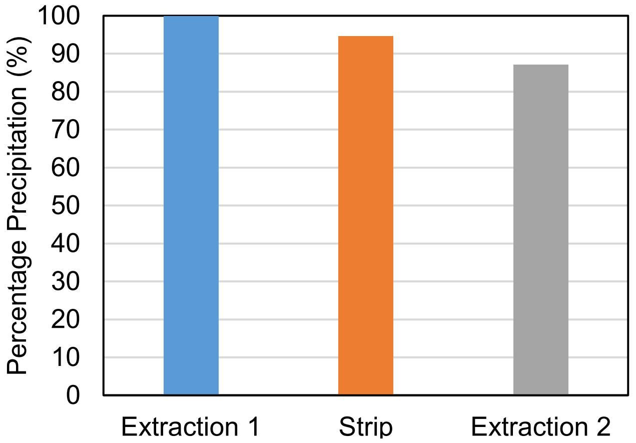

Supplementary Information Figure 13. Precipitation of lanthanum nitrate by L from 8 M HNO_3 (Extraction 1) follow by its release using methanol (strip) and the recovery of L with deionised water for its use in Extraction 2.

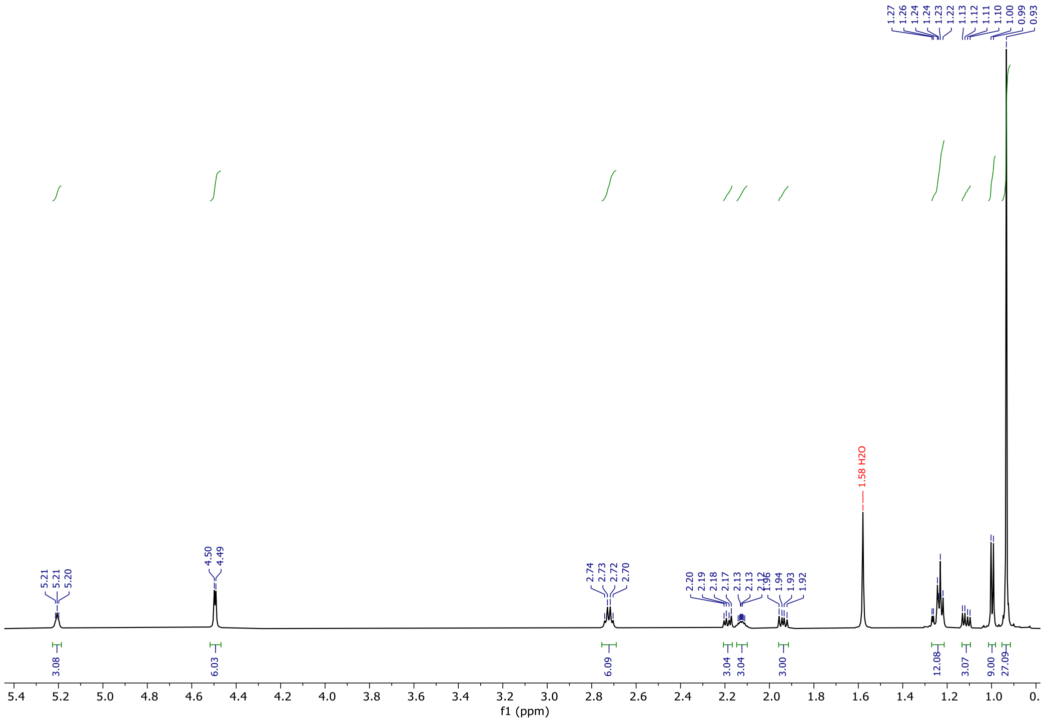

Supplementary Information Figure 14. ^{1}H NMR spectrum of the tripodal amido-arene L in $CDCl_3$.

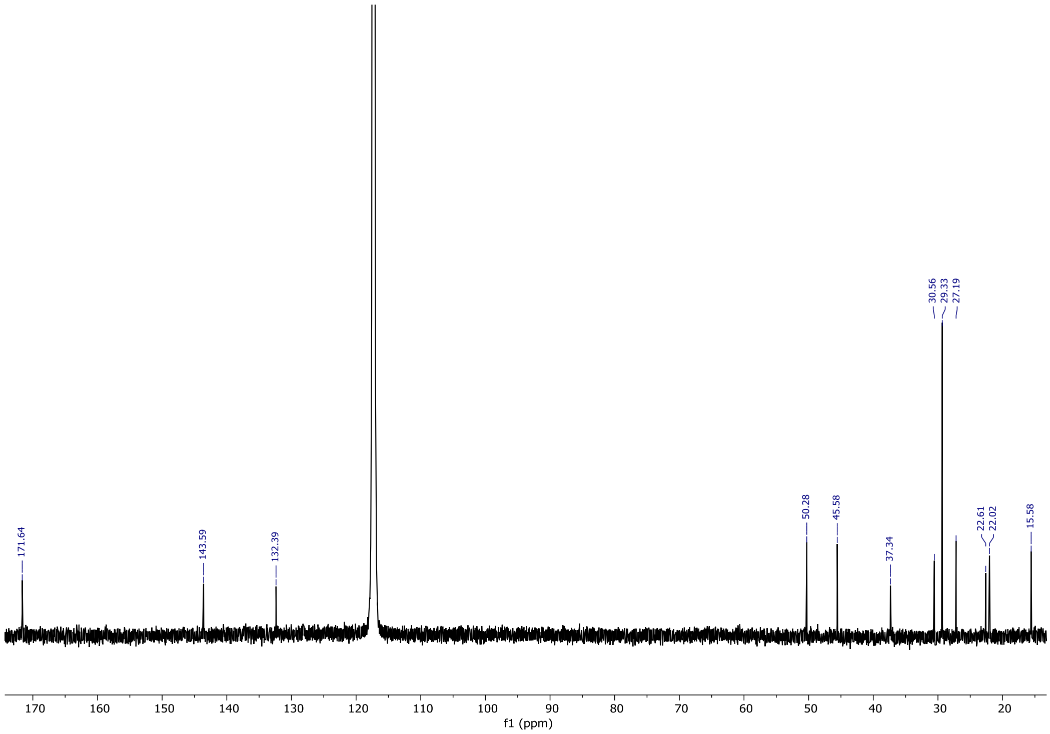

Supplementary Information Figure 15. $^{13}C\{^{1}H\}$ NMR spectrum of the tripodal amido-arene L in CD_3CN.

Supplementary Information Table 1. Crystal data for **1-La**

Crystal data	
Chemical formula	$LaN_6O_{18}\cdot C_{84}H_{153}N_6O_6$
M_r	1854.08
Crystal system, space group	Trigonal, *R*3
Temperature (K)	120
a, *c* (Å)	21.2897 (3), 20.4144 (3)
V (Å^3)	8013.2 (3)
Z	3
Radiation type	Cu *K*α
μ (mm^{-1})	3.64
Crystal size (mm)	0.13 × 0.09 × 0.07
Data collection	
Diffractometer	Rigaku Oxford Diffraction SuperNova
Absorption correction	Multi-scan<br>*CrysAlis PRO* 1.171.41.99a (Rigaku Oxford Diffraction, 2021) Empirical absorption correction using spherical harmonics, implemented in SCALE3 ABSPACK scaling algorithm.
T_{min}, T_{max}	0.550, 1.000
No. of measured, independent and observed [$I > 2\sigma(I)$] reflections	28366, 3701, 3677
R_{int}	0.075
$(\sin \theta/\lambda)_{max}$ (Å^{-1})	0.629
Refinement	
$R[F^2 > 2\sigma(F^2)]$, $wR(F^2)$, S	0.043, 0.116, 1.04
No. of reflections	3701
No. of parameters	252
No. of restraints	134
H-atom treatment	H atoms treated by a mixture of independent and constrained refinement
$\Delta\rho_{max}$, $\Delta\rho_{min}$ (e Å^{-3})	0.68, −0.82
CCDC number	2142978
